# An Investigation of Behavioural and Self-Reported Cognitive Empathy Deficits in Adolescents With Autism Spectrum Disorders and Adolescents With Behavioural Difficulties

**DOI:** 10.3389/fpsyt.2021.717877

**Published:** 2021-12-17

**Authors:** Sara P. Vilas, Renate L. E. P. Reniers, Amanda K. Ludlow

**Affiliations:** ^1^School of Psychology, University of Birmingham, Birmingham, United Kingdom; ^2^Department of Psychology, Universidad Europea de Madrid, Madrid, Spain; ^3^Institute of Clinical Science, University of Birmingham, Birmingham, United Kingdom; ^4^Institute for Mental Health, University of Birmingham, Birmingham, United Kingdom; ^5^School of Psychology, Sports and Geography, University of Hertfordshire, Hatfield, United Kingdom

**Keywords:** cognitive empathy, callous-unemotional traits, empathic accuracy, perspective taking, autism spectrum disorders, behavioural difficulties

## Abstract

Deficits in empathy have been considered hallmarks in individuals with autism spectrum disorders (ASD) but are also considered to underlie antisocial behaviour associated with individuals with callous unemotional traits (CU). Research has suggested that individuals with autism spectrum disorders show more difficulties with cognitive empathy, and that individuals diagnosed with behaviours difficulties, characterised by CU traits and antisocial behaviour, demonstrate low affective empathy. In the current manuscript we present findings of two studies. The first study describes the validation of a new stimulus set developed for the empathic accuracy task, focused on its cognitive component. The second study compares the performance of 27 adolescents with ASD, 27 age matched typically developing adolescents and 17 adolescents with behavioural difficulties on the empathic accuracy task and a self-report measure of empathy. While, no differences were observed between the three groups across the empathy accuracy task, the adolescents with ASD and CD showed deficits in their cognitive empathy across the self-report measure. Adolescents with ASD showed lower scores in particularly their perspective taking abilities, whereas the adolescences with behavioural difficulties showed more difficulties with their online simulation. No differences in self-reported affective empathy across the three groups were observed. Clinical implications of the findings are discussed.

## Introduction

Empathy is considered a multidimensional construct that is often as difficult to define as it is to measure. One common accepted definition features around the ability to be perceptive to and sympathetically experience the feelings of other people (affective empathy), while at the same time being able to put together a blueprint of their emotional states (cognitive empathy) (1). The importance of empathy is particularly apparent in disorders on the autism spectrum, where the ability to form social relationships and communicate with others is impaired (2). In addition, empathy is equally crucial in conduct disorders, which are characterized by reduced responsiveness to the distress of others in association with callous-unemotional traits (3). While both disorders are thought to be characterized by problems in empathy, social interaction and adaptation, these disorders reflect distinct problems in relationship to others (4). However, to date there has been little research comparing the two disorders directly on this construct.

It has been widely accepted that individuals with ASD have deficits in cognitive empathy (5–7), including lower levels of self-reported perspective taking (8) and poorer performance than typically developing adolescents on perspective taking tasks (9). Although evidence has generally shown a deficit in the processing of facial emotions [see (10) for a review], a meta-analysis has highlighted substantial inconsistencies between studies (11).

In terms of affective empathy, evidence is still mixed. Some studies have reported lower levels of cognitive and affective empathy (12), with deficits in the former component being more prominent than in the latter (13). Others have found deficits in cognitive empathy but not in affective empathy (7, 14, 15). Alternative theories have suggested that affective empathy is not impaired but heightened, and that it is this intensified ability which leads individuals with ASD to see the social world as more challenging and overwhelming (16).

Neurocognitive models suggest that the double dissociation on cognitive and affective aspects of empathy observed in ASD, is also present in other clinical disorders characterised by the manifestation of disruptive behaviours. This group of externalising disorders, known as Disruptive Behaviours Disorders (DBD), is characterised by a failure in the process of socialization as well as by oppositional, aggressive, rule-breaking and antisocial behaviours, and includes both Oppositional Defiant Disorder (ODD) and Conduct Disorder (CD) (17). In individuals with these conditions, a basic dysfunction in the affective component of empathy represents a core feature. For example, individuals with CD show poor capacities for affective resonance toward others' emotions, lack of concern for others' welfare (17–19) and lower levels of self-reported affective empathy (20, 21). Likewise, individuals with DBD have been found to exhibit lower levels of affective empathy as well as deficits in facial reactivity to angry expressions (22) and reduced heart rate reactivity in response to sadness (23, 24). Although individuals with CD (19) are thought to have intact cognitive empathy, evidence is still mixed. For example, Bons et al. (25) underlined in their review the mixed results in relation to emotion recognition (cognitive aspect of empathy), bringing into question whether cognitive empathy is truly preserved in CD. Some studies have reported reduced emotion recognition (26), while others have failed to find impairments in this ability (27).

It is important to mention that among individuals with CD, those with high levels of Callous Unemotional traits (CU) show a more severe and stable pattern of antisocial behaviour (28, 29), with a number of distinct social-cognitive deficits [see (30)]. Both classification systems, the Diagnostic and Statistical Manual of Mental Disorders, 5th Edition [DSM-5 (31)] and the International Classification of Disease, 11th Revision (ICD-11) (32), now have a specifier “with Limited Prosocial Emotions (LPE),” to refer to a group of children who have high levels of CU traits. CU traits are being identified by a lack of empathy, guilt, and being largely concerned about performance on important activities at the superficial level (33). The presence of CU traits amongst children has been found to be stable in those showing antisocial behaviour and these children are explicitly identified by decreased emotional reactivity to others' distress and lower sensitivity to punishment (34). Importantly, even without the presence of serious conduct problems, children with CU show high levels of interpersonal problems (35, 36).

While some studies have revealed a negative relationship between CU traits and both affective and cognitive empathy (30, 37), others have associated CU traits with deficits in cognitive but not affective empathy in females (38). In individuals with CD/psychopathic tendencies and high levels of CU traits, evidence has more consistently shown deficits in affective but not in cognitive empathy (19, 39).

Although both ASD and the above-mentioned disruptive behaviours are commonly referred to as empathy dysfunction disorders (40), evidence reveals that difficulties in empathy differ qualitatively among individuals with these conditions and hence, it should not be viewed simply as a global deficit. However, limited studies have investigated cognitive and affective empathy of adolescents with disruptive behaviours compared to those with ASD, and thus, the extent to which specific forms of empathy are associated with each disorder remains unclear. The available evidence, although still limited, has shown that boys with ASD only exhibit deficits in cognitive aspects of empathy (i.e., perspective taking), while those with psychopathic tendencies only show deficits in areas associated with affective empathy (39). In agreement with these results, Schwenck et al. (19) found that boys with ASD had impairments in perspective taking and showed a delay in the recognition of sad expressions, whereas children with CD and high CU were less emotionally affected when watching the scenes of the video sequences task, thereby reflecting a deficit in affective empathy. In this study, no deficits were observed in CD either for emotion recognition or for perspective taking. In addition, Bons et al. (25) found in their review that individuals with ASD also had impaired, or at least delayed, facial mimicry in response to static expressions for basic emotions (i.e., deficit in affective empathy), while adolescents with CD and high CU traits showed impaired emotion recognition for sad expressions (i.e., cognitive empathy deficit).

One of the core difficulties in assessing empathy in clinical populations is due to the favoured measurement of questionnaires such as the Empathising Quotient. However, items are often deemed vague and too imprecise, as well as being too focused on another's perception of your competence (41, 42). Experimental measures of empathy may play a vital role in illuminating the true nature of empathy (43).

Zaki et al. (44, 45) developed the Empathy Accuracy (EA) task to measure individuals' accurate inferences about the specific content of others' thoughts and feelings (46, 47). This task involves the use of social stimuli displaying realistic social interactions to investigate EA (i.e. cognitive empathy). This ability is defined as an intersubjective phenomenon that occurs between two people (47) and requires the ability to correctly judge or infer other's internal states (45). More specifically, EA refers to the ability of perceivers (individuals who observe another person) to notice, attend, and correctly interpret the observable behaviours of social targets (individuals who are the focus of the perceivers' attention). These behaviours are transmitted by the targets through facial expressions, voice tone and/or words, and translated by the perceivers into inferences about targets' internal states, i.e., thoughts and emotions (48, 49). There are two main aspects involved in EA. The first aspect, known as content accuracy, refers to the degree to which the perceivers' inferences about the content of targets' internal states matches the actual content of targets' internal states. The second aspect, valence accuracy, refers to the degree by which the perceivers' inferences about the emotional tone (positive, neutral, negative) of targets' internal states matches the actual valence of targets' internal states (47).

Although people often attempt to infer others' thoughts and feelings in their daily interactions (a process known as empathic inference), it is the extent to which such attempts are successful that is classified as EA (50). Therefore, within social interaction contexts, EA is considered an essential aspect of empathy, as it helps guide social behaviour (49) and avoid/ reduce conflicts with others (51), thereby contributing to successful social interactions and facilitating social adjustment (48). Recent research has revealed however that perceivers' EA may rely more on the extent to which targets' behaviour reflects their internal states, rather than on features of the perceivers (44). Indeed, evidence has shown that emotional expressivity predicts EA when targets use more intense and frequent facial expressions or affective language, i.e., visually exhibiting more negative affect or verbally expressing more positive affect (45).

A strong link between EA and autism has been proposed within the Theory of Mind (ToM) framework, where individuals with ASD are considered as being mind-blind or unable to accurately infer others' thoughts and feelings (52, 53). This corresponds with empirical studies showing that both adolescents and adults with pervasive developmental disorders (PDD) or ASD are able to infer others' thoughts and feelings when the situation observed is more predictable and less complex (i.e., structured conversation). However, they perform worse than controls when greater communicative and social abilities are required (i.e., less structured conversation) (54, 55).

The majority of research addressing EA in DBD has focused around the addition of CU traits (3). While those with CU traits have problems with emotional reactivity to distress cues and are therefore associated with an affective deficit, there is also the assumption that those with CU carry less problems reported for cognitive empathy and related constructs, such as perspective-taking, emotion recognition, and ToM. However, a recent metanalysis in adults with CU traits found difficulties in both cognitive and affective empathy (56).

In the original Empathic Accuracy Task (EA task), Zaki et al. examined the relationships between perceivers' trait measures of empathy and their empathic accuracy and found that perceivers' trait affective empathy was unrelated to empathic accuracy when targets were low in expressivity. Only more recently have researchers incorporated another component to the EA task to allow affective empathy to also be assessed behaviourally, here requesting participants to report whether they share the depicted emotion. These studies have found no differences in either cognitive or affective empathy using the behavioural measure in adults with ASD compared to a group of typically developing adults. However, some deficits were noted on the cognitive empathy self-report questionnaire (57). When the behavioural measure was assessed in adolescents with conduct disorder, affective empathy deficits were reported (58). It deserves a critical note, however, if asking participants to report whether they share the depicted emotion is a true measure of affective empathy or whether it is muddled by cognitive components involving construction of a working model of another's and one's own emotional states. Therefore, the current study only assessed cognitive empathy in the AE task.

In the current manuscript we present findings of two studies. The first study aimed to develop a new stimulus set for a behavioural measure of empathy, focused on its cognitive component, using the EA task protocol previously used by Zaki et al. (44, 45). Similar to the Zaki study, cognitive aspects of empathy and empathic accuracy were examined using the EA task and compared to self-reported levels of affective and cognitive empathy. The second study aimed to extend the research in empathy deficits in ASD and individuals with DBD by examining cognitive and affective empathy abilities using the EA and self-report empathy measures in both clinical populations when compared to a control group of typically developing adolescents. Due to the limited access to adolescents with a formal diagnosis of DBD, a broader group of adolescents with emotional and behavioural difficulties (BD) was recruited for the present study. The developmental period of adolescence constitutes a period of great physical health, yet also a period during which onset of severe mental illness peaks. It is a formative period during which young people develop greater independence while being subjected to increases in affective reactivity that come with greater vulnerability to emotional (and behavioural) dysregulation (59). We suggest that empathy plays an important role in young people's lives, helping them to regulate their emotions and make sense of the social world they live in. Because of this importance, our study focussed on the adolescent age.

We predicted the following: firstly, ASD and BD were expected to have lower levels of EA than controls, with difficulties in this task being specific to the inference of negative emotions, as shown by previous studies focused on emotion recognition (25, 26). We also hypothesised that individuals with ASD would report lower levels of cognitive empathy with difficulties in perspective taking being specific to the ASD group. In contrast, individuals with BD would show lower levels of trait affective empathy (19, 39). Finally, we predicted the BD group to have higher levels of CU traits than both ASD and controls.

## Materials and Methods -Study 1: Development and Validation of New Stimuli For The EA Task

### Participants

#### Targets

Sixteen (7 males, 9 females; Mage = 19.02 years, SD = 0.61), originally took part in the study. The majority identified as being white British (87.5%, *n* = 14; Asian-Indian = 12.5%, *n* = 2). Following ratings by perceivers (see procedure below), videos were removed due to lack of emotional expressivity by the target, film and sound quality. This left videos from only 10 of the original participants, aged between 18 and 20 (5 males, 5 females; Mage = 18.96 years, SD = 0.58), with the majority identifying as being either white English (80%) and Asian-Indian (20%).

#### Perceivers

Fifty-nine university students (50 females, 9 males) aged between 18 and 32 years old (Mage= 21 years and 6 months; SD 3.43) were recruited to rate the videos. They performed the task individually in a laboratory. Both the targets and perceivers were students in psychology courses at universities in the United Kingdom. They were all unpaid volunteers and completed the ratings for course credits.

### Materials and Procedure for Assessing Empathic Accuracy

The EA Task was adapted from (44, 60). There were two phases: In the initial target phase, we created videos of young adult participants (Targets) discussing emotional events in their lives. After watching their own videos targets rated how positive or negative, they had felt while speaking. In the subsequent perceiver phase, an unrelated group of young adult perceivers watched these videos and continuously rated how they thought the target was feeling during each video. Our measure of EA was the r-to-z-transformed correlation between perceivers' ratings of targets' feelings and targets' ratings of their own feelings. Both phases of the study were conducted according to the principles expressed in the Declaration of Helsinki and were approved by the Ethics Committee of the University of < city>Birmingham </city>, United Kingdom. All participants provided written informed consent before the completion of the measures and after having received information about the study (e.g., voluntary participation, confidentiality/anonymity, right to withdraw) and the research team, and all questions were answered satisfactorily. Participants (Targets) included in the videos of the EA task provided written consent not only for them to be filmed, but also for the films to be watched by young adults (Perceivers). More detailed information on each of the phases of task development:

#### Phase 1

Participants were asked to recall and list four positive and four negative autobiographical events that they were comfortable describing and willing to discuss in front of a camera. They were asked to write a brief description about these events, in addition to providing them with a title (a maximum length of five words), and to rate the emotional valence and intensity of each event by using a 9-points Likert scale that ranged from 1 (very negative) to 9 (very positive). Only events with a certain grade of emotional burden, i.e., those rated by the target as having an emotional intensity above the scale's midpoint, were included in the discussion phase. For each participant, the researcher pseudorandomised the order of the events to be discussed, alternating events with positive valence with those with negative valence, as previously described by (44). After removal of 15 events that were rated by the target as having an emotional intensity below the scale's midpoint, 113 events were included in the subsequent discussion stage. The Targets were given the list of events to be discussed and were seated facing the camera directly, with the frame capturing them from the shoulders up. They were then asked to describe the event and discuss the details and emotions experienced. After discussing each event with no time limit, targets were asked to rate the valence and intensity of the emotions they had experienced while discussing and remembering each event using a Likert scale ranging from 1 (very negative) to 9 (very positive). These ratings were referred to as affective ratings. The selection of these videos was done as follows. A total 30 videos were excluded because the targets rated (after discussing the events) their own emotions as having an averaged or neutral intensity, 5 videos were excluded due to poor sound quality, and 24 videos were excluded because the targets were not directly facing the camera when discussing the events. The final 16 videos were chosen taking into consideration: (1) the valence of the videos, with half of the videos describing negative events and the other half describing positive events; (2) gender of the targets (8 males; 8 females); (3) length of the videos (M = 64.94; minimum video length = 20 s, maximum video length = 1 min and 46 s) and (4) the content of the videos, in order to avoid repetition of topics.

#### Phase 2

Perceivers were asked to complete the EA task on a desktop computer that ran the E-Prime experiment displayed on a 22.6″ monitor. Participants were asked to continuously rate how positive or negative they believed the target of each video was feeling at each moment by using the left or right arrow keys to move along a 9-point scale. Detailed instructions on how to complete the task were verbally provided prior to the completion of the task. Then, perceivers were asked to watch and rate two practice videos that did not form part of the pool of videos included in the EA task. However, both practice videos matched the videos from the EA task on length and affective ratings. There were no significant differences in the length of the videos (including the practice videos) based on targets' gender, *t*_(16)_ = −0.33, *p* = 0.75, or the valence of the events, *t*_(16)_ = 0.55, *p* = 0.59, nor in targets' affective ratings based on their gender, *t*_(16)_ = 0.56, *p* = 0.59. After this, perceivers were presented with the set of videos included in the EA task. This involved watching 16 videos (8 positive and 8 negative) in a pseudorandomised order that ensured that the visualisation of the positive videos was alternated with that of the negative ones, and that the order of the presentation for the videos was different for each participant. Furthermore, the presentation of the videos was split across four runs, which allowed participants to rest between each run.

### Measures of Emotion (Completed by Both Targets and Perceivers*)*

#### Emotion Regulation Questionnaire

ERQ (61) is a self-report questionnaire with 10 items rated on a scale of 1 (strongly disagree) to 7 (strongly agree) that assesses the tendency to regulate emotions by means of two strategies. The first, cognitive reappraisal, refers to the ability to reduce the emotional impact of a situation by changing the way we interpret it (62). The second, expressive suppression, is defined as the intentional inhibition of our emotional expressive behaviour when observing emotional stimuli (63). Satisfactory psychometric properties were found in the present study, with Cronbach's α of 0.82 for cognitive reappraisal and 0.78 for expressive suppression.

#### Berkeley Expressivity Questionnaire

BEQ (64) is a self-report questionnaire with 16 items rated on a 7-point-likert scale (ranging from strongly disagree to strongly agree). It assesses three aspects of emotional expressivity: negative and positive expression of emotions, and impulse strength. Negative expressivity refers to the expression of emotions such as anger, fear, nervousness, and upset, while positive expressivity includes, for example, warmth and friendliness. Impulse strength refers to the difficulty to control strong emotional impulses (64, 65). Cronbach's α of 0.74 were found in the present study.

#### Analyses Strategy

All analyses were performed using SPSS Version 20 (IBM SPSS Inc., Armonk, NY). A significance alpha level of 0.05, and two-tailed tests were used for statistical analyses. The first study was intended to develop the EA task. Data reduction, i.e., extraction of targets' and perceivers' reaction times and affective ratings, were done using E-prime. Time-series correlations were performed as follows. Continuous affective ratings were converted into a time-series of sequential values, with one value for each second period. These values served as data points in subsequent time series analyses. Targets and perceivers' affective ratings were z-transformed across the entire session to correct for interindividual variation in the use of the rating scale. To calculate the EA of participants, perceivers' continuous affective ratings were correlated with the targets' own continuous ratings, by using Pearson's correlations. The resulting correlation coefficient (r) between two time-series was the measure of EA. This coefficient was calculated separately for each perceiver-video combination. Correlation coefficients were r-to-z transformed by performing Fisher transformations in preparation for subsequent analyses.

## Results

Importantly, no significant differences were found between perceivers and targets in self-reported levels of emotion regulation [cognitive reappraisal, *t*_(67)_ = −0.48, *p* = 0.63, expressive suppression, *t*_(67)_ = −0.33, *p* = 0.74], or emotional expressivity, *t*_(67)_ = 1.17, *p* = 0.25. No significant differences were found on levels of cognitive reappraisal, expressive suppression, or expressivity between male and female targets.

The perceivers were accurate when rating targets' affect (*M* = 0.62, SD = 0.12), with EA coefficients ranging between 0.21 and 0.92. There were no significant differences in young adult accuracy in distinguishing positive events (*M* = 0.60, SD = 0.14; EA range: 0.06–0.74) from negative events *(M* = 0.62, *SD* = 0.12; EA range: 0.20–0.81) *t*_(116)_ = −0.60, *p* = 0.55. There were no significant differences in EA between male (*M* = 0.60, SD = 0.16) and female (*M* = 0.62, SD = 0.11) perceivers, *t*_(57)_ = 0.41, *p* = 0.68, although female perceivers showed in general higher EA. There were also no significant differences between male and female perceivers when assessing videos either with males or females (Table 1).

**Table 1:** Means, standard deviations and *p*-values comparing EA based on perceivers' gender.

		***M***	***SD***	***P***
Videos with female targets	Female perceivers	0.54	0.09	0.80
	Male perceivers	0.53	0.11	
Videos with male targets	Female perceivers	0.68	0.14	0.75
	Male perceivers	0.66	0.23	

*Two-separated t-test analyses based on the gender of the targets were conducted to investigate gender differences on perceivers' EA*.

Results showed that there was no effect of perceivers' levels of cognitive empathy (β = −0.06, *t* = −0.39, *p* = 0.70) or affective empathy (β = 0.15, *t* = 1.05, *p* = 0.30) on their EA [R2 = 0.02, ΔR2 = −0.02, *F*_(2,56)_ = 0.55, *p* = 0.58]. Targets' levels of negative expressivity (β = 0.14, *t* = 0.35, *p* = 0.74) did not significantly predict perceivers' EA for videos with negative valence, R2 = 0.02, ΔR2 = −0.14, *F*_(1,6)_ = 0.13, *p* = 0.74. For videos with positive valence, targets' levels of positive expressivity (β = 0.88, *t* = 4.54, *p* < 0.01) were found to be a significant predictor of perceivers' EA, R2 = 0.78, ΔR2 = 0.74, *F*_(1,6)_ = 20.65, *p* < 0.01. Targets' levels of emotional expressivity were not significantly correlated with the intensity of the affect ratings of their own videos, *r*_(16)_ = 0.17, *p* = 0.54.

Results showed that neither perceivers' levels of cognitive reappraisal (β = −0.05, *t* = −0.41, *p* = 0.69) nor expressive suppression (β = 0.06, *t* = 0.43, *p* = 0.67) significantly predicted perceivers' EA for videos with positive valence, R*2* = 0.01, *F*_(2,56)_ = 0.19, *p* = 0.82. Likewise, for videos with negative valence, neither perceivers' levels of cognitive reappraisal (β = 0.05, *t* = −0.37, *p* = 0.71) nor expressive suppression (β = −0.23*, t* = −1.76, *p* = 0.08) were found to be significant predictors of perceivers' EA, *R2* = 0.05, ΔR2 = 0.02, *F*_(2,56)_ = 1.57, *p* = 0.22.

Previous research has shown AE coefficients ranging from 0.46 (60) and 0.47 (44, 45, 66) to 0.52 (67) and as high as 0.68 (68). Our AE coefficient of 0.62 falls within this range. Whilst empathy levels of targets and perceivers have been reported to have no impact on AE (44, 68), high expressivity scores of targets seem to positively impact AE (44, 45, 67). This is consistent with the findings in the current study.

## Materials and Methods: Study 2: Empathy in children With and Without ASD and BD

### Participants

Seventy-one participants (37 males, 34 females) aged between 12 and 17 (*Mage* = 15.26 years, *SD* = 1.28) took part in the study. Three groups of participants were recruited from secondary schools in the West Midlands, United Kingdom. For the first group, the control group (CG), 27 typically developing individuals (7 males, 20 females) were recruited from one academy sponsor-led (*n* = 3) and two comprehensives (*n* = 25). For the second group, ASD, a total 27 participants (23 males, 4 females) with ASD were included. Participants were recruited from one specialist foundation for individuals with special educational needs (SEN); one specialist school for individuals with SEN, in which a formal diagnosis of autism was the criterion for entry; and one independent day school for people with a formal diagnosis of an Autism Spectrum Disorder, and one school for people with formal diagnosis of autism referred by the Child and Adolescent Mental Health Service were also included. For the third group, a total of 17 participants with BD (7 males, 10 females) were included. Participants were recruited from one specialist foundation for individuals with a diagnosis of social, emotional and mental health needs; one pupil referral unit for students who have been permanently excluded from school; one community centre for adolescents experiencing social, behavioural and emotional difficulties; and one converter academy for girls with SEN. Eligibility criteria included capacity to provide informed consent and fluency in English to be able to complete all the measures. All the typically developing children were required to have no known neurodevelopmental disorders such as autism, attention deficit disorder or behavioural disorder as reported by both parents and confirmed via school records. Differences between groups on demographics characteristics were examined, revealing a significant between group effect on age, *F*_(2,68)_ = 6.21, *p* < 0.01, with participants with BD being significantly younger (Mage = 14.39) than both participants with ASD (Mage = 15.67, *p* < 0.01) and controls (Mage = 15.39, *p* < 0.05). No significant differences were found in age between participants with ASD and controls (*p* = 0.67). The three groups also differed by gender, χ2 _(1, N = 71)_ = 20.07, *p* < 0.001, with the number of females being significantly higher in the control group (7 males, 20 females) and BD group (7 males, 10 females). In the ASD group, the number of males was significantly higher than the females (23 males, 4 females).

Only individuals who had a formal diagnosis of any of the following conditions: Asperger's Syndrome, ASD, or PDD-NOS as confirmed by both the parents and school, were able to take part. All the participants from this group reported having been diagnosed with either ASD (85%, *n* = 23) or Asperger's Syndrome (15%, *n* = 4). Participants were aged between 3 and 15 (Mage = 7.52 years, SD = 3.65) when diagnosed, and these diagnoses were made by psychiatrists (41%), the Child and Adolescent Mental Health Service (CAHMS) (26%), psychologists (18%), or paediatricians (15%). According to the school records children had no recognised intellectual disability. Ten participants reported the co-occurrence of one or more co-morbid disorders, including ADHD (*n* = 3), obsessive-compulsive disorder (*n* = 2), dyspraxia (*n* = 4), dyslexia (*n* =1), dyscalculia (*n* = 1) and general learning difficulties (*n* = 1).

For the BD group, selection criteria included (1) no-presence of co-morbid clinical diagnosis of a neurodevelopmental disorder and (2) attendance to specialist institutions to which entry was dependent upon the manifestation of BD. Specific clinical diagnoses for the children were not made available by the schools, so it was unclear how many children identified as having clinical DBD such as CD, oppositional Defiant Disorder (ODD), and those meeting more subclinical levels. The Youth Psychopathic Traits Inventory [YPT: Andershed et al. (69)] was completed by participants from the BD group to confirm the presence of BD. The YPI is a 50-item self-report questionnaire that assesses traits of psychopathic personality on interpersonal, affective, and behavioural domains. It has shown satisfactory psychometric properties (Cronbach's α coefficient of 0.93 in the present study). Participants from the BD group reported, in general, increased levels of psychopathic features (*M* = 2.64, SD = 0.55, minimum = 1.84, maximum = 3.44), with 8 out of 12 participants scoring on the YPT above the proposed cut-off (i.e., 2.5 out of 4) to define those who score high on psychopathic traits (70).

The Inventory of Callous–Unemotional Traits [ICU; Frick (71)] and the Antisocial Process Screening Device [APSD; Frick and Hare (72)] were administered to further characterise the BD group in comparison to the ASD and control groups. The ICU is a 24-items self-report questionnaire rated on a four-point scale from 0 (not at all true) to 3 (definitely true) that assesses three aspects of CU traits: uncaring, callous, and unemotional traits. These traits reflect, in addition to the lack of empathy, lack of guilt and poverty in emotional expression. Only the self-report version of the ICU was used in the current study due to the limited access to participants' parents, as some of them came from home backgrounds where parental non-response was considered highly likely. This questionnaire demonstrated moderate to good reliability (with Cronbach's α coefficients ranging from 0.45 to 0.88 for its three subscales) and good construct validity in schools (73) and among adolescent offenders (74, 75). This questionnaire has shown satisfactory psychometric properties in the present study, with a Cronbach's α coefficient of 0.58, 0.75, and 0.81 for the unemotional, callousness and uncaring subscales, respectively. The APSD is a 20-items brief report questionnaire rated on a three-point scale: 0 (not at all true), 1 (sometimes true), 2 (definitely true) that assesses several aspects of antisocial behaviour, including narcissism, CU, and impulsivity traits. A self-report version of the APSD has been developed for older youths (between 12 and 18 years), and this has been suggested to be a more reliable and valid measure of antisocial features among adolescents. In addition, this questionnaire has been shown to have good reliability and validity (72). This questionnaire has shown satisfactory psychometric properties in the present study, with an overall Cronbach's α coefficient of 0.78.

The combination of the ICU and the APSD provides a comprehensive assessment of callous and unemotional traits (76), which is important to define a distinct subgroup group of antisocial and aggressive youth, thereby allowing for the classification of participants within a subgroup of individuals with behavioural difficulties in the present study.

### Measures of Empathy

#### Questionnaire of Cognitive and Affective Empathy

QCAE (1) is a questionnaire with 31-items rated on a scale of 1 (strongly disagree) to 4 (strongly agree) that assesses self-reported levels of cognitive and affective empathy. The first refers to the ability to build a working model of others' emotions whereas the second involves being sensitive to and vicariously experiencing others' feelings (1). The cognitive scale is made up of two subcomponents: *Perspective taking* which involves intuitively putting oneself in another person's shoes to see things from his or her perspective and *online simulation* which encompasses an effortful attempt to put oneself in another person's position by imagining what that person is feeling. Online simulation is likely to be used for future intentions. The affective scale is made up of three components: *Emotion contagion* assesses the automatic mirroring of the feelings of others. *Proximal responsivity*, addresses the responsiveness aspect of empathic behaviour, illustrated by the affective response when witnessing the mood of others in a close social context. Similar to proximal responsivity but in a detached context is *peripheral responsivity*. The QCAE has clear factor structure, good reliability and verified convergent and construct validity (1). This questionnaire has shown satisfactory psychometric properties in the first (Cronbach's α of 0.88 for cognitive empathy and 0.83 for affective empathy) and second study (Cronbach's α of 0.83 for cognitive empathy and 0.68 for affective empathy).

#### Empathic Accuracy Task: A Measure of Behavioural Cognitive Empathy

The computerised experiment adapted from (44, 60) and described above was used to assess participants' EA, which is defined as the ability to judge others' expressive behaviour centred on the words spoken, tone of voice and also on one's facial expressions. For the purpose of this study, the EA was adapted to create a shorter version to reduce burden on the participants (the task was predicted to be challenging for the ASD and BD groups), and this was administered to all the participants. For this short version, 12 videos were chosen taking into consideration valence of the events (6 positive; 6 negative), gender of the targets (6 males; 6 females), and length of the videos (*M* = 65.5; Range = 37 s; 1 min and 37 s). Although there were no significant differences in the length of the videos based on targets' gender, *t*_(10)_ = −0.86, *p* = 0.41, significant differences were found in the length of the videos based on valence of the events described, *t*_(10)_ = −3.39, *p* < 0.01. Negative videos were found to be significantly longer (*M* = 79.5, SD = 17.97) than positive ones (*M* = 51.5, SD = 9.31).

### Procedure

The study was conducted according to the principles expressed in the Declaration of Helsinki and were approved by the Ethics Committee of the University of < city>Birmingham </city>, United Kingdom. Written and verbal consent was obtained from all the children included in the study along with their parent/legal guardian/carer's written consent after having received information about the study (e.g., voluntary participation, confidentiality/anonymity, right to withdraw) and the research team, and all questions were answered satisfactorily. After providing informed consent as outlined above, all participants were asked to complete socio-demographic questions and then presented with three self-report questionnaires assessing empathy and symptoms questionnaires in a fixed order (i.e., QCAE, ICU and APSD). These were completed in a quiet room during one-to-one sessions with the researcher of 20–35 min, giving them extra time to complete the measures if required. Subsequently, participants were asked to complete the EA task, which lasted approximately 15 min. Participants could take breaks as often as they needed.

### Analyses Strategy

Correlations between self-reported levels of cognitive and effective empathy and levels of EA were investigated. We also examined differences in empathy and antisocial/ CU traits between ASD, BD, and controls using multivariate analysis. Parametric analyses were conducted due to normality of the data. *Post-hoc* analyses using Bonferroni corrections were conducted. In the results section, adjusted *p*-values were reported. Considering the significant between group effect found on age, *F*_(2,68)_ = 6.21, *p* < 0.01, and gender, χ2_(1, N = 71)_ = 20.07, *p* < 0.001, between the BD group, participants with ASD and controls; both age and gender were used as covariates of interest.

## Results

### CU and Antisocial Traits in ASD and BD

One-way MANCOVA analysis revealed an overall significant effect on CU traits (callousness, uncaring and unemotional), *F*_(6,128)_ = 2.74, *p* < 0.05; Wilk's Λ = 0.79, ηp2 = 0.11, across groups, after controlling for age and gender. Subsequent univariate ANOVAs analysis showed significant differences in callousness, *F*_(2,66)_ = 6.99; MSE = 171.69; *p* < 0.01; ηp2 = 0.18, and uncaring, *F*_(2,66)_ = 3.57; MSE = 75.54; *p* < 0.05; ηp2 = 0.10, but not in unemotional traits, *F*_(2,66)_ = 1.90; MSE = 16.26; *p* = 0.16; ηp2 = 0.05, with BD reporting significantly higher levels than ASD and controls. Bonferroni *post hoc* analysis revealed that only levels of callous traits were significantly higher in BD than ASD (*p* < 0.05) and controls (*p* < 0.01). No significant differences were found between ASD and controls (*p* > 0.05). See Table 2 for descriptive statistics.

**Table 2:** Means (standard deviations) on CU and antisocial traits, and their subscales.

	**CG (*n* = 27)**	**ASD (*n* = 27)**	**BD (*n* = 17)**
CU traits (ICU)	24.33 (7.83)	24.74 (8.61)	35.06 (11.54)
Callousness	7.22 (4.06)	9.19 (4.80)	12.76 (6.40)*
Uncaring	8.26 (3.56)	8.00 (4.52)	12.94 (6.28)*
Unemotional	8.85 (3.33)	7.56 (2.24)	9.35 (3.18)
Antisocial traits (APSD)	11.41 (4.73)	13.37 (5.83)	18.65 (4.69)
Narcissism	3.37 (2.56)	4.22 (2.61)	5.18 (2.23)*
Impulsivity	3.78 (1.37)	4.44 (1.70)*	6.12 (1.45)***
CU traits	3.37 (1.55)	3.70 (1.88)	5.65 (2.18)***

*p < 0.05; **p < 0.01; ***p < 0.001. All significant differences were in comparison controls.

A second one-way MANCOVA analysis also showed an overall significant effect on antisocial traits (narcissism, impulsivity and CU traits), *F*_(6,128)_ = 4.29, *p* < 0.01; Wilk's Λ = 0.69, ηp2 = 0.17, across groups, after controlling for age and gender. Following univariate ANOVAs analysis confirmed the significant differences in narcissism, *F*_(2,66)_ = 3.09; MSE = 18.90; *p* < 0.05; ηp2 = 0.09, impulsivity, *F*_(2,66)_ = 10.89; MSE = 23.58; *p* < 0.001; ηp2 = 0.25, as well as CU traits, *F*_(2,66)_ = 6.36; MSE = 21.83; *p* < 0.01; ηp2 = 0.16. Bonferroni *post hoc* analysis with adjusted significance revealed that levels of narcissism (*p* < 0.05), impulsivity (*p* < 0.001) and CU traits (*p* < 0.01) were significantly higher in BD than controls. *Post hoc* analysis showed that levels of impulsivity were significantly higher in ASD than controls (*p* < 0.05). No significant differences were found between ASD and BD (*p* > 0.05). Levels of CU traits were significantly lower in ASD than BD (*p* < 0.05). No significant differences were found between ASD and controls (*p* > 0.05).

#### Empathic Accuracy in ASD and BD

One-way MANCOVA analyses were conducted to investigate the differences between ASD, BD, and controls in EA and each of its subtypes. Multivariate analysis showed no overall effect on EA and each of its subtypes, *F*_(10,114)_ = 1.48, *p* = 0.15; Wilk's Λ = 0.78, ηp2 = 0.12, after controlling for age and gender. EA coefficients are shown in Table 3.

**Table 3:** EA coefficients for each group.

		**EA coefficients**			
**Group**	***N***	**Minimum**	**Maximum**	**Mean**	**Std dev**
Control	26	0.37	0.69	0.57	0.088
ASD	24	0.09	0.71	0.53	0.16
BD	17	−0.03	0.69	0.47	0.20

Separate analysis of variance revealed a significant difference in EA for videos with female targets between ASD and controls, *F*_(1,46)_ = 4.20; MSE = 0.21; *p* < 0.05; ηp2 = 0.08, after controlling for gender. Participants with ASD showed lower levels of EA for videos with female targets (*M* = 0.40, SD = 0.31) than controls (*M* = 0.49, SD = 0.12). Significant differences were also found in EA based on type of the event described in both controls, *t*_(50)_ = 04.18, *p* < 0.001, and ASD, *t*_(46)_ = 2.73, *p* < 0.01. All the perceivers were more accurate at assessing positive than negative events (Table 3).

#### Self-Reported Empathy in ASD and BD

One-way ANCOVA analyses were conducted to study the differences between ASD, BD, and controls in self-reported levels of cognitive and affective empathy, after controlling for age and gender. Significant differences were found between the three groups of participants in cognitive empathy, *F*_(2,66)_ = 3.05; *MSE* = 219.42; *p* < 0.05; ηp2 = 0.09, but not in affective empathy, *F*_(2,66)_ = 2.23; *MSE* = 66.55; *p* = 0.12; ηp2 = 0.06. Differences between ASD, BD, and controls in all the subcomponents of cognitive empathy were further investigated. Multivariate analysis showed an overall significant effect on both perspective taking and online simulation (components of cognitive empathy), *F*_(4,134)_ = 2.60, *p* < 0.05; Wilk's Λ = 0.86, ηp2 = 0.07. Subsequent univariate ANOVAs analysis showed significant differences in online simulation, *F*_(2,68)_ = 3.63; MSE = 95.29; *p* < 0.05; ηp2 = 0.10, but not in perspective taking, *F*_(2,68)_ = 2.84; MSE = 65.05; *p* = 0.07; ηp2 = 0.08. Bonferroni *post hoc* analysis with adjusted significance revealed that levels of online simulation were significantly lower in BD than controls (*p* < 0.05). No significant differences were found between adolescents with ASD and those with BD (*p* = 0.66) or controls (*p* = 0.37). See Table 4 for descriptive statistics.

**Table 4:** Means (standard deviations) for cognitive and affective empathy, and their subscales.

	**CG (*n* = 27)**	**ASD (*n* = 27)**	**BD (*n* = 17)**
Cognitive empathy (QCAE)	56.30 (7.67)*	51.11 (7.11)*	49.76 (10.91)*
- Perspective taking	30.96 (4.46)	27.96 (3.48)*	28.65 (6.73)
- Online simulation	25.33 (5.19)	23.15 (4.79)	21.12 (5.52)*
Affective empathy (QCAE)	33.15 (6.11)	31.07 (4.23)	29.12 (6.26)
- Emotion contagion	10.41 (2.99)	10.11 (1.93)	10.00 (4.24)
- Proximal responsivity	11.81 (2.80)	11.33 (2.13)	10.29 (2.89)
- Peripheral responsivity	10.93 (2.06)	9.63 (2.12)	8.82 (2.90)

*p < 0.05; **p < 0.01; ***p < 0.001. All significant differences were in comparison to controls.

One-way MANCOVA analyses were carried out to investigate the differences between participants with and without ASD in all the subcomponents of cognitive empathy. Results showed an overall significant effect on both perspective taking and online simulation, *F*_(2,50)_ = 3.96, *p* < 0.05; Wilk's Λ = 0.86, ηp2 = 0.14. Subsequent univariate ANOVAs analysis revealed a significant difference in perspective taking, *F*_(1,51)_ = 7.09; MSE = 114.54; *p* < 0.01; ηp2 = 0.12, with ASD reporting lower levels than controls (see Table 5). No significant differences were found in online simulation, *F*_(1,51)_ = 3.34; MSE = 83.59; *p* = 0.07; ηp2 = 0.06.

**Table 5:** Descriptive statistics for the videos selected for the development of the EA task-short version.

**Targets'**	**Valence**	**Topic**	**Video length**	**Mean (SD) of**
**gender**	**of event**		**(in seconds)**	**affective ratings**
Female	Positive	Seeing a boyfriend	50	6.43 (1.51)
Female	Positive	A level grade	37	7.00 (1.00)
Male	Positive	Weight loss	47	7.00 (1.00)
Female	Positive	Birth of youngest brother	52	6.50 (0.71)
Male	Positive	Emily's Birthday	61	7.00 (1.00)
Male	Positive	Kittens	62	6.80 (0.84)
Female	Negative	Losing the pub/home	78	4.17 (0.75)
Female	Negative	Break up	52	5.30 (1.34)
Female	Negative	Visiting grandma	94	4.43 (0.98)
Male	Negative	The NewCom fallout	97	3.83 (1.47)
Male	Negative	Parent's divorce	91	3.00 (1.00)
Male	Negative	Beatty's ill health	65	3.00 (1.00)

*Affective ratings refer to the continuous ratings made by targets when watching*.

## Discussion

The current work set out to examine and directly compare cognitive and affective empathy abilities in adolescents with ASD, BD, and typically developing adolescents. In order to assess cognitive empathy on both the behavioural and self-report level, the first study validated a new stimulus set for the EA task (44, 45). This task was used in the second study to investigate the ability of clinical populations to accurately assess others' emotional states, using social stimuli that depicted male and female targets experiencing real emotions. The second study furthermore compared group performance on self-report measures of CU traits, antisocial behaviour, and empathy. The presence of higher levels of CU traits and antisocial behaviour characterise the BD group as having overt behavioural difficulties but may have shown milder symptomatology than expected if all the children met a clinical diagnosis for certain DBD such as CD. The adolescents with ASD showed marked deficits in their cognitive empathy, for self-report measures only. Adolescents with ASD showed lower scores in particularly their perspective taking abilities, whereas the adolescents with BD showed more difficulties with their online simulation. No significant differences in affective empathy across the three groups were observed.

Four key findings were obtained for the development and validation of the EA task. First, there were no significant differences in EA between male and female perceivers, although females tended to show higher EA than males. Unexpectedly, targets' gender was found to be as significant predictor of perceivers EA, with perceivers being more accurate at assessing male targets' emotions. Second, perceivers' EA was not influenced by their own self-reported levels of cognitive and affective empathy. Third, positive expressivity of targets was found to be a significant predictor of perceivers' EA, showing the perceivers an increased EA for highly expressive targets. In contrast, negative expressivity of targets did not predict perceivers' EA. Lastly, contrary to our expectations, levels of emotion regulation (either from targets or perceivers) were not associated with perceivers' EA.

Taken together, these results suggest that EA depends more on specific characteristics of the target (i.e., gender and positive expressivity) than on those of the perceiver (i.e., gender, trait cognitive and affective empathy). The literature has previously shown no significant differences between male and female perceivers in EA (50, 77), and our results provided further support for this idea. Interestingly, our results also showed that perceivers (both males and females) were more accurate at assessing male targets' affect than female targets' affect. This finding seems to contradict previous evidence suggesting that because females are more expressive than males (65), their emotions should be easier to be inferred compared to those from male targets (78). However, the fact that females usually report themselves as being not only more expressive, but also more ambivalent in their emotional expressions compared to males (79), could explain why emotional expressions from males were more accurately inferred.

Our results demonstrated the significance of emotional expressivity for EA, showing that targets' positive emotional expressivity predicted EA when perceivers assessed targets' affect from positive videos. Our findings suggest that emotions from targets with higher levels of positive expressivity are easier to be perceived and accurately inferred by perceivers. This supports, to some extent, prior work indicating that targets' emotional expressivity predicts perceivers' EA (44). Our results suggest an asymmetry in the accurate inference of others' internal states based on the valence of the expressed emotion, indicating that positive emotional expressions could be considered as visually more distinctive and recognisable than the negative ones. In fact, evidence has revealed an advantage in the processing of positive facial expressions compared to negative expressions. In terms of speed of recognition, positive facial expressions (e.g., happiness) have been found to be recognised faster than negative expressions (e.g., disgust or sadness) (80, 81). Considering the accuracy of emotion recognition, happy facial expressions have been more accurately recognised than negative expressions (i.e., disgust, anger and sadness), even when positive expressions have a relatively low intensity (82), or when these are presented unexpectedly under conditions in which negative facial expressions are unnoticeable (83). Furthermore, positive expressions are less likely to be misjudged as neutral expressions due to the manifestation of characteristic features, such as a smile, that can be used as precise indicative cues (80). The current study successfully developed a new stimulus set for the EA task. The use of this task will allow measuring EA as a performance variable, thereby providing our research with a viable alternative to avoid the limited ecological validity associated with the use of pictures tasks in the assessment of empathy features in clinical and non-clinical populations.

The second study aimed to compare the performance of 27 adolescents with ASD, 27 matched typically developing adolescents and 17 adolescents with BD on the behavioural EA task and self-report measure of empathy. As expected, individuals with ASD performed worse in the EA task than matched controls, although these differences were statistically significant only when measuring EA for videos with female targets. Our results also showed that the control participants were more accurate at assessing male targets' affect than female targets' affect. The fact that females usually report themselves as being not only more expressive, but also more ambivalent in their emotional expressions compared to males (79) could explain why emotional expressions from males were more accurately inferred. This matches our findings in study 1. Likewise, differences in EA based on type of the event described were found in both control and ASD participants, with both groups of perceivers assessing more accurately positive than negative events. As mentioned above, our results suggest that positive emotional expressions are more easily inferred than negative expressions because positive expressions seem to be visually more distinctive and recognised faster than negative expressions (80, 81).

Similar to previous studies addressing EA in adolescents with CD, no differences were found for the BD group across cognitive empathy on the EA task (84). In addition to the behavioural measure of empathy, a self-report questionnaire was administered to further assess cognitive and affective components of empathy. Our results revealed that levels of self-reported affective empathy did not significantly differ across groups, although reported levels of affective empathy were lower in BD than ASD, with controls having the higher scores. The results are suggestive of others reported in ASD literature, suggesting their affective empathy to be intact (85, 86). Furthermore, the lack of deficit in affective empathy found in the adolescents with BD mirrors those of Robinson and Rogers (87), who also failed to find differences in affective empathy when comparing three groups of offenders with different levels of psychopathic traits. However, this finding disagrees with individuals meeting a clinical diagnosis of a disruptive behaviour disorder, with lower levels of self-reported affective empathy found in this group (21, 22). This may suggest that the “milder” symptomatology experienced by our BD group compared to individuals with DBD may be associated with intact vs. impaired levels of affective empathy and warrants further research to explore possible causal associations between severity of symptomatology and levels of affective empathy in these individuals.

The lack of differences in affective empathy could also be related to the type of items used in each questionnaire. While the affective items from the empathy questionnaire (QCAE) focused more on the experience of emotions and affective responses, the items assessing lack of empathy as part of CU traits (ICU) seem to be more related to behaviours. Seeing that individuals with disruptive behaviours show poor capacities for affective resonance toward others' emotions, it is possible that they misjudge their own affective responses on the QCAE (e.g., “It pains me to see young people in wheelchairs”), but accurately assess their behavioural responses when completing the ICU (“I apologise to persons I hurt”).

In contrast, significant differences were found in self- reported cognitive empathy across groups, with individuals with BD reporting significantly more difficulties than controls. Our results contradict previous studies that have failed to find difficulties in cognitive empathy in individuals with samples displaying antisocial behaviour compared to controls (19, 88). They also disagree, to some extent, with the proposed double dissociation of empathy, in which individuals with ASD tend to display more deficits in cognitive than affective empathy (7, 13–15), while those with disruptive behaviours show the opposite profile (17, 19, 39, 89).

Examining the differences across groups in the subcomponents of cognitive empathy we found that levels of online simulation were lower in individuals with BD than in both controls and individuals with ASD. Differences between ASD and BD were, however, not statistically significant. In addition, there is a negative correlation between chronological age and impulsivity, with the latter declining significantly from childhood through adolescence and into adulthood (90). Seeing that our sample included participants aged between 12 and 17, it may be the case that the deficits observed in cognitive empathy (i.e., online simulation) will not be present in the group of participants with BD in later developmental stages. This corresponds with research revealing that boys with psychopathic traits tend to exhibit analogous levels of cognitive empathy than their peers, suggesting that the observed deficits in this ability may not persist after adolescence (38).

As predicted, adolescents with ASD reported significantly lower scores on cognitive empathy than the controls, while no differences were found in affective empathy between both groups. Our findings concur with those of previous studies supporting a dissociation between cognitive and affective empathy by using self-report questionnaires (7, 15). Furthermore, the literature has consistently shown the existence of a perspective taking deficit in ASD (8, 91), and our results provide further support for this idea. Our findings also mirror those using the EA task in adults with ASD, showing no differences in cognitive on the EA, but some cognitive empathy deficits on the self- report questionnaires, namely perspective taking (57). In summary, our results show a cognitive deficit in ASD that seems to be specific to the subcomponent of perspective taking, and suggests that adolescents with ASD seem to have, at least to a certain extent, insight into their poor perspective taking abilities (55).

Examining the subcomponents of cognitive empathy further, we found that levels of online simulation (i.e., an attempt to put oneself in others' place by imagining what that person is feeling) (1), were lower in individuals with BD than in both controls and individuals with ASD. Differences between ASD and BD were, however, not statistically significant. This is consistent with findings by (87), who found that offenders with high psychopathy traits display lower levels of online simulation than offenders with medium and low psychopathy traits. The authors suggested that their findings could be explained the fact that online simulation measures the active effort to put oneself in another's place through their imagination rather than using a more analytic perspective, such as the self-assessment of their own ability. Considering that online simulation often refers to future intentions (e.g., “Before criticising somebody, I try to imagine how I would feel if I was in their place”), difficulties within this ability could also be explained by the frequent co-occurrence between impulsivity/ behavioural disinhibition and disruptive behaviour disorders (92–94). Perhaps the impulsive behaviour associated with these conditions (95, 96) leads individuals to quickly respond to a given situation rather than to evaluate (e.g., by using online simulation) this situation first. Indeed, our results showed that impulsivity (as measured by the APSD) was higher in individuals with BD when compared to both ASD and controls (although the differences between BD and ASD were not statistically significant).

Finally, we found that individuals with BD reported higher levels of CU traits than those with ASD and controls. In particular, its subcomponent callousness was found to be significantly higher in BD than ASD and controls, reflecting a lack of guilt and empathy within those with BD (97). These results provide, to some extent, support for evidence revealing that individuals with disruptive behaviours have a basic dysfunction in affective empathy that is characterised by poor capacities for affective resonance toward others' emotions and lack of concern for others' welfare (17–19). Corresponding with previous research (98), individuals with ASD reported an increase in callousness traits compared to controls (although these differences were not statistically significant), suggesting a potential selective deficit in affective domains that includes the ability to care about others' feelings. It is worth noting that according to literature, the presence of CU traits in ASD seems to be more associated with behavioural features characteristic of ASD, such as lack of sensitivity to the feelings of others, rather than with the manifestation of conduct problems (99). In fact, our results showed that levels of CU traits were significantly lower in individuals with ASD when compared to those within the BD group.

Although the current findings are promising, there are also some limitations to be noted. Firstly, there were gender differences across groups. Due to low prevalence of ASD among females, we mainly included males in the ASD group, whereas in the BD and control groups the number of females included was higher than the number of males. While the gender imbalance should be considered a limitation of the study and generalisability of the results, previous studies have found no gender differences on the EA task in neither clinical nor non-clinical adolescent populations (44, 67, 84). For example, no gender differences have been found on the EA in typically developing adolescents and those with CD (58, 100). Kral et al. (68) did find a significant effect of gender in typically developing adolescents with female having higher AE coefficients, however, this effect dropped to trend level when controlling for age. Equally, given that the ASD group may have performed worse on the EA task for female targets as male participants are generally worse at EA of female targets, it is important to note that no differences have been reported in empathic behaviours, both in style and levels, between adolescent females and males with and without ASD (86). As participants in the current study constituted of adolescents, generalisation of the findings to adult populations should be cautioned.

Amongst the ASD group, it should be noted that three children had a comorbid diagnosis with ADHD, and these children may have performed different to those with ASD. These 3 children were not removed from the analysis as these disorders often co-occur (101). Research has also failed to find differences between child adolescents with ASD compared to those with ADHD using an EA task (54). Furthermore, none of the children included in the study were identified as having an intellectual disability; this should be verified with appropriate assessments in future research. It is also important to mention that we were not able to recruit individuals with a formal diagnosis of CD, BDB, or related conduct problems, and therefore our results need to be interpreted more in line of individuals who show higher levels of behavioural difficulties but may not meet the thresholds required for a formal clinical diagnosis. However, all the participants with BD attended special schools, which ensured a pattern of behavioural problems. This was further demonstrated by the predicted scores on the CU and ASPD measures. It would be of interest to extend this research to clinical samples of adolescents comparing different clinical diagnoses of DBD (e.g. ODD vs. CD).

Finally, the EA task in the current study focused on the cognitive aspect of empathy only, which meant that a direct comparison could not be made on affective empathy using behavioural and self-report measures. The Multifaceted Empathy Test (MET) captures cognitive and emotional components of empathy within the same task and has been shown to be a useful and efficient instrument for indexing impaired empathy in different diagnostic groups and may therefore be useful to include alongside the EA task in future studies addressing both clinical groups (14, 102).

Overall, our findings revealed no overall deficit in empathy as highlighted by the EA task in any of the three groups. Rather, the results support the existence of a deficit in cognitive empathy in ASD, which seemed to be specific to the perspective taking subcomponent, and suggest the preservation of their affective empathy, thereby supporting the double dissociation proposed for both components of empathy. In addition, our findings provide evidence of a cognitive deficit in empathy, in particular online simulation, in individuals with BD that could be better explained by the demographic characteristics of our sample (i.e., age of participants with BD; non-clinical levels of BD). Although both ASD and the above-mentioned disruptive behaviours are commonly referred to as empathy dysfunction disorders (40), our results reveal that difficulties in cognitive empathy differ qualitatively among individuals highlighting it should not be viewed simply as a global deficit.

## Data Availability Statement

Summary statistics are available on request by the first author Sara P. Vilas, sarapaloma.vilas@universidadeuropea.es.

## Ethics Statement

The studies involving human participants were reviewed and approved by Ethics Committee of the University of Birmingham, United Kingdom. Written informed consent to participate in this study was provided by the participants' legal guardian/next of kin. In addition, participants also provided written and verbal consent.

## Author Contributions

The study was designed by SV under supervision of AL and RR. SV collected the data. SV conducted the data analysis under guidance of AL and RR. All authors contributed to the article and approved the submitted version.

## Funding

SV studies were supported by a Hilary Green Studentship on empathy awarded by the University of Birmingham, United Kingdom.

## Conflict of Interest

The authors declare that the research was conducted in the absence of any commercial or financial relationships that could be construed as a potential conflict of interest.

## Publisher's Note

All claims expressed in this article are solely those of the authors and do not necessarily represent those of their affiliated organizations, or those of the publisher, the editors and the reviewers. Any product that may be evaluated in this article, or claim that may be made by its manufacturer, is not guaranteed or endorsed by the publisher.

## References

[1] Reniers RL Corcoran R Drake R Shryane NM Völlm BA. The QCAE: a questionnaire of cognitive and affective empathy. J Person Assess. (2011) 93:84–95. 10.1080/00223891.2010.528484 21184334

[2] Decety J Moriguchi Y. The empathic brain and its dysfunction in psychiatric populations: implications for intervention across different clinical conditions. Bio Psycho Soc Med. (2007) 1:1–21. 10.1186/1751-0759-1-22 18021398 PMC2206036

[3] Frick PJ Kemp EC. Conduct disorders and empathy development. Annu Rev Clin Psychol. (2021). 17:391–416. 10.1146/annurev-clinpsy-081219-105809 33290109

[4] Vilas Sanz S Ludlow A Reniers R. Empathy dysfunction: deconstructing social functioning in autism spectrum disorders and conduct disorder. In: Watt DF, Panksepp J, editors. Psychology and Neurobiology of Empathy. NOVA Science Publishers, Inc. (2016).

[5] Grove R Baillie A Allison C Baron-Cohen S Hoekstra RA. The latent structure of cognitive and emotional empathy in individuals with autism, first-degree relatives and typical individuals. Mol Autism. (2014) 5:1–10 10.1186/2040-2392-5-42 PMC4123248 25101164

[6] Mathersul D McDonald S Rushby JA. Understanding advanced theory of mind and empathy in high-functioning adults with autism spectrum disorder. J Clin Exp Neuropsychol. (2013) 35:655–68. 10.1080/13803395.2013.809700 23799244

[7] Rueda P Fernández-Berrocal P Baron-Cohen S. Dissociation between cognitive and affective empathy in youth with asperger syndrome. Eur J Dev Psychol. (2015) 12:85–98. 10.1080/17405629.2014.950221

[8] Hirvelä S Helkama K. Empathy, values, morality and asperger's syndrome. Scand J Psychol. (2011) 52:560–72. 10.1111/j.1467-9450.2011.00913.x 21967087

[9] Tibbetts PA Rehfeldt RA. Assessing relational learning deficits in perspective-taking in children with high-functioning autism. Behav Dev Bulletin. (2005) 12:62–8. 10.1037/h0100562

[10] Harms MB Martin A Wallace GL. Facial emotion recognition in autism spectrum disorders: a review of behavioral and neuroimaging studies. Neuropsychol Rev. (2010) 20:290–322. 10.1007/s11065-010-9138-61 20809200

[11] Uljarevic M Hamilton A. Recognition of emotions in autism: a formal meta-analysis. J Autism Dev Dis. (2013) 43:1517–26. 10.1007/s10803-012-1695-5 23114566

[12] Shamay-Tsoory SG Tomer R Yaniv S Aharon-Peretz J. Empathy deficits in asperger syndrome: a cognitive profile. Neurocase. (2002) 8:245–52. 10.1093/neucas/8.3.245 12119321

[13] Mazza M Pino MC Mariano M Tempesta D Ferrara M De Berardis D. Affective and cognitive empathy in adolescents with autism spectrum disorder. Front Human Neurosci. (2014) 8:1–6. 10.3389/fnhum.2014.00791 25339889 PMC4187579

[14] Dziobek I Rogers K Fleck S Bahnemann M Heekeren HR Wolf OT. Dissociation of cognitive and emotional empathy in adults with asperger syndrome using the multifaceted empathy test (MET). J Autism Dev Dis. (2008) 38:464–73. 10.1007/s10803-007-0486-x 17990089

[15] Rogers K Dziobek I Hassenstab J Wolf OT Convit A. Who cares? Revisiting empathy in asperger syndrome. J Autism Dev Dis. (2007) 37:709–15. 10.1007/s10803-006-0197-8 16906462

[16] Markram H Rinaldi T Markram K. The intense world syndrome–an alternative hypothesis for autism. Front Neurosci. (2007) 1:77–96. 10.3389/neuro.01.1.1.006.2007 18982120 PMC2518049

[17] de Wied M Goudena PP Matthys W. Empathy in boys with disruptive behavior disorders. J Child Psychol Psychiatry. (2005) 6:867–80. 10.1111/j.1469-7610.2004.00389.x 16033635

[18] Green J Gilchrist A Burton D Cox A. Social and psychiatric functioning in adolescents with asperger syndrome compared with conduct disorder. J Autism Dev Dis. (2000) 30:279–93. 10.1023/A:1005523232106 11039855

[19] Schwenck C Mergenthaler J Keller K Zech J Salehi S Taurines R. Empathy in children with autism and conduct disorder: group-specific profiles and developmental aspects. J Child Psychol Psychiatry. (2012) 53:651–9. 10.1111/j.1469-7610.2011.02499.x 22118246

[20] Cohen D Strayer J. Empathy in conduct-disordered and comparison youth. Dev Psychol. (1996) 32:988–98. 10.1037/0012-1649.32.6.98832735912

[21] Lovett BJ Sheffield RA. Affective empathy deficits in aggressive children and adolescents: a critical review. Clin Psychol Rev. (2007) 27:1–13. 10.1016/j.cpr.2006.03.003 16697094

[22] de Wied M van Boxtel A Zaalberg R Goudena PP Matthys W. Facial EMG responses to dynamic emotional facial expressions in boys with disruptive behavior disorders. J Psychiatric Res. (2006) 40:112–21. 10.1016/j.jpsychires.2005.08.003 16176819

[23] de Wied M Boxtel AV Posthumus JA Goudena PP Matthys W. Facial EMG and heart rate responses to emotion-inducing film clips in boys with disruptive behavior disorders. Psychophysiology. (2009) 46:996–1004. 10.1111/j.1469-8986.2009.00851.x 19549069

[24] de Wied M van Boxtel A Matthys W Meeus W. Verbal, facial and autonomic responses to empathy-eliciting film clips by disruptive male adolescents with high versus low callous-unemotional traits. J Abnor Child Psychol. (2012) 40:211–23. 10.1007/s10802-011-9557-8 21870040 PMC3267933

[25] Bons D Broek E Scheepers F Herpers P Rommelse N Buitelaaar J. Motor, emotional, and cognitive empathy in children and adolescents with autism spectrum disorder and conduct disorder. J Abnor Child Psychol. (2013) 41:425–43. 10.1007/s10802-012-9689-5 23096764

[26] Fairchild G Stobbe Y van Goozen SHM Calder AJ Goodyer IM. Facial expression recognition, fear conditioning, and startle modulation in female subjects with conduct disorder. Biol Psychiatry. (2010) 68:272–9. 10.1016/j.biopsych.2010.02.019 20447616 PMC2954286

[27] Pajer K Leininger L Gardner W. Recognition of facial affect in girls with conduct disorder. Psychiatry Res. (2010) 175:244–51. 10.1016/j.psychres.2009.06.003 20022121

[28] Frick PJ Ray JV Thornton LC Kahn RE. Can callous-unemotional traits enhance the understanding, diagnosis, and treatment of serious conduct problems in children and adolescents? A comprehensive review. Psychol Bulletin. (2014) 140:1–57. 10.1037/a0033076 23796269

[29] Frick PJ White SF. Research review: the importance of callous-unemotional traits for developmental models of aggressive and antisocial behavior. J Child Psychol Psychiatry. (2008) 49:359–75. 10.1111/j.1469-7610.2007.01862.x 18221345

[30] Pardini DA John EL Paul JF. Callous/unemotional traits and social-cognitive processes in adjudicated youths. J Am Acad Child Adol Psychiatry. (2003) 42:364–71. 10.1097/00004583-200303000-00018 12595791

[31] American Psychiatric Association. Diagnostic and Statistical Manual of Mental Disorders. 5th ed. Washington, DC: American Psychiatric Association (2013).

[32] World Health Organization. International Statistical Classification of Diseases and Related Health Problems (11th Revision). World Health Organization (2018). Retrieved from: https://icd.who.int/browse11/l-m/en

[33] Kemp EC Frick PJ Matlasz TM Clark JE Robertson EL Ray JV. Developing cutoff scores for the inventory of callous-unemotional traits (ICU) in justice-involved and community samples. J Clin Child Adol Psychol. (2021) 1–14. 10.1080/15374416.2021.1955371 34424103

[34] Blair RJR Leibenluft E Pine DS. Conduct disorder and callous-unemotional traits in youth. N Engl J Med. (2014) 371:2207–16. 10.1056/NEJMra1315612 25470696 PMC6312699

[35] Graziano PA Ros R Haas S Hart K Slavec J Waschbusch D. Assessing callous-unemotional traits in preschool children with disruptive behavior problems using peer reports. J Clin Child Adol Psychol. (2016) 45:201–14. 10.1080/15374416.2014.971460 25587855

[36] Matlasz TM Frick PJ Clark JE. Understanding the social relationships of youth with callous-unemotional traits using peer nominations. J Clin Child Adol Psychol. (2020) 1–13. 10.1080/15374416.2020.1823847 33125288

[37] Pasalich DS Dadds MR Hawes DJ. Cognitive and affective empathy in children with conduct problems: additive and interactive effects of callous–unemotional traits and autism spectrum disorders symptoms. Psychiatry Res. (2014) 219:625–30. 10.1016/j.psychres.2014.06.025 25015711

[38] Dadds MR Hawes DJ Frost AD Vassallo S Bunn P Hunter K. Learning to ‘talk the talk': the relationship of psychopathic traits to deficits in empathy across childhood. J Child Psychol Psychiatry. (2009) 50:599–606. 10.1111/j.1469-7610.2008.02058.x 19445007

[39] Jones AP Happ é FG Gilbert F Burnett S Viding E. Feeling, caring, knowing: different types of empathy deficit in boys with psychopathic tendencies and autism spectrum disorder. J Child Psychol Psychiatry. (2010) 51:1188–97. 10.1111/j.1469-7610.2010.02280.x 20633070 PMC3494975

[40] Blair JR. Responding to the emotions of others: dissociating forms of empathy through the study of typical and psychiatric populations. Consci Cogn. (2005) 14:698–718. 10.1016/j.concog.2005.06.004 16157488

[41] Edey R Cook J Brewer R Johnson MH Bird G Press C. Interaction takes two: Typical adults exhibit mind-blindness towards those with autism spectrum disorder. J Abnorm Psychol. (2016). 125:879–85. 10.1037/abn0000199 27583766

[42] Sheppard E Pillai D Wong GTL Ropar D Mitchell P. How easy is it to read the minds of people with autism spectrum disorder?. J Autism Dev Dis. (2016) 46:1247–54. 10.1007/s10803-015-2662-8 26603886

[43] Fletcher-Watson S Bird G. Autism and empathy: what are the real links? Autism. (2020) 24:3–6. 10.1177/1362361319883506 31674189

[44] Zaki J Bolger N Ochsner K. It takes two the interpersonal nature of empathic accuracy. Psychol Sci. (2008) 19:399–404. 10.1111/j.1467-9280.2008.02099.x 18399894

[45] Zaki J Bolger N Ochsner K. Unpacking the informational bases of empathic accuracy. Emotion. (2009) 9:478–87. 10.1037/a0016551 19653768 PMC6558959

[46] Ickes W. Empathic accuracy. J Pers. (1993) 61:587–610.

[47] Ickes WJ Stinson L Bissonnette V Garcia S. Naturalistic social cognition: empathic accuracy in mixed-sex dyads. J Person Soc Psychol. (1990) 59:730–42. 10.1037/0022-3514.59.4.730

[48] Gleason KA Jensen-Campbell LA Ickes WJ. The role of empathic accuracy in adolescents' peer relations and adjustment. Person Soc Psychol Bulletin. (2009) 35:997–1011. 10.1177/0146167209336605 19498068

[49] Zaki J Ochsner K. Reintegrating the study of accuracy into social cognition research. Psychol Inquiry. (2011) 22:159–82. 10.1080/1047840X.2011.551743

[50] Ickes WJ. Empathic Accuracy. New York, NY: Guilford Press (1997).

[51] Simpson JA Ickes WJ Orina M. Empathic accuracy pre-emptive relationship maintenance. In: Harvey J, Wenzel A, editors. Close Romantic Relationships: Maintenance and Enhancement. Mahwah, NJ: Lawrence Erlbaum (2001). p. 27–46.

[52] Baron-Cohen S. Mindblindness: An Essay on Autism and Theory of Mind. Cambridge, MA: Bradford Books, MIT Press (1997).

[53] Ickes WJ. Empathic accuracy: its links to clinical, cognitive, developmental, social and physiological psychology. In: Decety J, Ickes W, editors. The Social Neuroscience of Empathy. Cambridge, MA: MIT Press (2009). p. 57–70. 10.7551/mitpress/9780262012973.003.0006

[54] Demurie E De Corel M Roeyers H. Empathic accuracy in adolescents with autism spectrum disorders and adolescents with attention-deficit/hyperactivity disorder. Res Autism Spec Dis. (2011) 5:126–34. 10.1016/j.rasd.2010.03.002

[55] Ponnet K Roeyers H Buysse A De Clercq A Van Der Heyden E. Advanced mind-reading in adults with asperger syndrome. Autism. (2004) 8:249–66. 10.1177/1362361304045214 15358869

[56] Waller R Wagner NJ Barstead MG Subar A Petersen JL Hyde JS. A meta-analysis of the associations between callous-unemotional traits and empathy, prosociality, and guilt. Clin Psychol Rev. (2020) 75:101809. 10.1016/j.cpr.2019.101809 31862383

[57] McKenzie K Russell A Golm D Fairchild G. Empathic accuracy and cognitive and affective empathy in young adults with and without autism spectrum disorder. J Autism Dev Dis. (2021) 1–5. 10.1007/s10803-021-05093-7 PMC9021079 34052970

[58] Martin-Key NA Allison G Fairchild G. Empathic accuracy in female adolescents with conduct disorder and sex differences in the relationship between conduct disorder and empathy. J Abnor Child Psychol. (2020) 48:1155–67. 10.1007/s10802-020-00659-y 32488571 PMC7392945

[59] Wood SJ Reniers RLEP Diaz-Arteche C Pantelis C. Adolescent brain development and implications for mental health. In: Yung AR, McGorry P, ediorss. Youth Mental Health: A Preventive Approach to Mental Disorders in Young People. Abingdon, VA: Taylor & Francis Group (2020).

[60] Zaki J Weber J Bolger N Ochsner K. The neural bases of empathic accuracy. Proc Natl Acad Sci USA. (2009) 106:11382–7. 10.1073/pnas.0902666106 19549849 PMC2708723

[61] Gross JJ John OP. Individual differences in two emotion regulation processes: implications for affect, relationships, and well-being. J Pers Soc Psychol. (2003) 85:348. 10.1037/0022-3514.85.2.348 12916575

[62] Gross JJ. Emotion regulation: Affective, cognitive, and social consequences. Psychophysiology. (2002) 39:281–91. 10.1017/S0048577201393198 12212647

[63] Gross JJ Levenson RW. Emotional suppression: physiology, self-report, and expressive behavior. J Pers Soc Psychol. (1993). 64:970–86. 10.1037/0022-3514.64.6.970 8326473

[64] Gross JJ John OP. Revealing feelings: facets of emotional expressivity in self-reports, peer ratings, and behavior. J Pers Soc Psychol. (1997) 72:435. 9107009 10.1037//0022-3514.72.2.435

[65] Gross JJ John OP. Facets of emotional expressivity: three self-report factors and their correlates. Person Ind Differen. (1995) 19:555–68. 10.1016/0191-8869(95)00055-B

[66] Meadors J. Rethinking Empathic Accuracy (Accession No. 6c5968c1) [Doctoral dissertation, University of California, Riverside]. UC Riverside Electronic Theses and Dissertations, Riverside, CA (2014).

[67] Van Donkersgoed RJM De Jong S Aan het Rot M. Measuring empathy in schizophrenia: the empathic accuracy task and its correlation with other empathy measures. Schizoph Res. (2019) 208:153–9. 10.1016/j.schres.2019.03.024 31006615

[68] Kral TRA Solis E Mumford JA Schuyler BS Flook L Rifken K. Neural correlates of empathic accuracy in adolescence. Soc Cogn Affect Neurosci. (2017) 12:1701–10. 10.1093/scan/nsx099 28981837 PMC5714170

[69] Andershed H Kerr M Stattin H Levander S. Psychopathic traits in non-referred youths: a new assessment tool. In: Blau E, Sheridan L, editors. Psychopaths: Current International Perspectives. Amsterdam: Elsevier (2002). p. 131–58.

[70] Skeem JL Cauffman E. Views of the downward extension: comparing the youth version of the psychopathy checklist with the youth psychopathic traits inventory. Behav Sci Law. (2003) 21:737–70. 10.1002/bsl.563 14696029

[71] Frick PJ. The Inventory of Callous-Unemotional Traits. New Orleans, LA: University of New Orleans (2004).

[72] Frick PJ Hare RD. Antisocial Process Screening Device (APSD): Technical Manual. Toronto, ON: MHS (2001).

[73] Essau CA Sasagawa S Frick PJ. Callous-unemotional traits in a community sample of adolescents. Assessment. (2006) 13:454–69. 10.1177/1073191106287354 17050915

[74] Kimonis ER Frick PJ Skeem JL Marsee MA Cruise K Munoz LC. Assessing callous-unemotional traits in adolescent offenders: validation of the Inventory of Callous-Unemotional Traits. Int J Law Psychiatry. (2008) 31:241–52. 10.1016/j.ijlp.2008.04.002 18514315

[75] Roose A Bijttebier P Decoene S Claes L Frick PJ. Assessing the affective features of psychopathy in adolescence: a further validation of the inventory of callous and unemotional traits. Assessment. (2010) 17:44–57. 10.1177/1073191109344153 19797326

[76] Byrd AL Kahn RE Pardini DA. A validation of the Inventory of Callous-Unemotional Traits in a community sample of young adult males. J Psychopathol Behav Assess. (2013) 35:20–34. 24357894 10.1007/s10862-012-9315-4 PMC3864815

[77] Ickes W Gesn PR Graham T. Gender differences in empathic accuracy: differential ability or differential motivation? Pers Relatsh. (2000) 7:95–109. 10.1111/j.1475-6811.2000.tb00006.x

[78] Klein KJ Hodges SD. Gender differences, motivation, and empathic accuracy: when it pays to understand. Person Soc Psychol Bulletin. (2001) 27:720–30. 10.1177/0146167201276007

[79] King LA Emmons RA. Conflict over emotional expression: psychological and physical correlates. J Person Soc Psychol. (1990) 58:864–77. 10.1037/0022-3514.58.5.864 2348373

[80] Calvo MG Marrero H. Visual search of emotional faces: the role of affective content and featural distinctiveness. Cogn Emot. (2009) 23:782–806. 10.1080/02699930802151654

[81] Leppänen JM Hietanen JK. Positive facial expressions are recognized faster than negative facial expressions, but why? Psychol Res. (2004) 69:22–9. 10.1007/s00426-003-0157-2 14648224

[82] Hess U Blairy S Kleck RE. The intensity of emotional facial expressions and decoding accuracy. J Nonv Behav. (1997) 21:241–57. 10.1023/A:1024952730333

[83] Mack A Rock I. Inattentional Blindness (Vol. 33). Cambridge, MA: MIT press (1998).

[84] Rum Y Perry A. Empathic accuracy in clinical populations. Front Psychiatry. (2020) 11:457. 10.3389/fpsyt.2020.00457 32581862 PMC7283465

[85] Mul CL Stagg SD Herbelin B Aspell JE. The feeling of me feeling for you: interoception, alexithymia and empathy in autism. J Autism Dev Dis. (2018) 48:2953–67. 10.1007/s10803-018-3564-3 29644587

[86] Rieffe C O'Connor R Bülow A Willems D Hull L Sedgewick F. Quantity and quality of empathic responding by autistic and non-autistic adolescent girls and boys. Autism. (2021) 25:199–209. 10.1177/1362361320956422 32967463 PMC7812514

[87] Robinson EV Rogers R. Empathy faking in psychopathic offenders: the vulnerability of empathy measures. J Psychopathol Behav Assess. (2015) 37:545–52. 10.1007/s10862-015-9479-9

[88] Dolan M Fullam R. Theory of mind and mentalizing ability in antisocial personality disorders with and without psychopathy. Psychol Med. (2004) 34:1093–102. 10.1017/S0033291704002028 15554579

[89] Blair JR. Fine cuts of empathy and the amygdala: dissociable deficits in psychopathy and autism. Quart J Exp Psychol. (2008) 61:157–70. 10.1080/17470210701508855 18038346

[90] Galvan A Hare T Voss H Glover G Casey B. Risk-taking and the adolescent brain: who is at risk? Dev Sci. (2007) 10:F8–14. 10.1111/j.1467-7687.2006.00579.x 17286837

[91] Castelli F Frith C Happé F Frith U. Autism, asperger syndrome and brain mechanisms for the attribution of mental states to animated shapes. Brain. (2002) 125:1839–49. 10.1093/brain/awf189 12135974

[92] Frick PJ Lilienfeld SO Ellis M Loney B Silverthorn P. The association between anxiety and psychopathy dimensions in children. J Abnorm Child Psychol. (1999) 27:383–92. 10.1023/a:1021928018403 10582839

[93] Milich R Hartung CM Martin CA Haigler ED. Behavioral disinhibition and underlying processes in adolescents with disruptive behavior disorders. In: Disruptive Behavior Disorders in Childhood. Boston, MA: Springer (1994). p. 109–38.

[94] Waschbusch DA. A meta-analytic examination of comorbid hyperactive-impulsive-attention problems and conduct problems. Psychol Bull. (2002) 128:118–50. 10.1037/0033-2909.128.1.118 11843545

[95] Avila C Parcet MA. Personality and inhibitory deficits in the stop-signal task: the mediating role of Gray's anxiety and impulsivity. Pers Individ Differ. (2001) 31:975–86. 10.1016/S0191-8869(00)00199-9

[96] Moeller FG Barratt ES Dougherty DM Schmitz JM Swann AC. Psychiatric aspects of impulsivity. Am J Psychiatry. (2001) 158:1783–93. 10.1176/appi.ajp.158.11.1783 11691682

[97] Frick PJ Viding E. Antisocial behavior from a developmental psychopathology perspective. Dev Psychopathol. (2009) 21:1111–31. 10.1017/S0954579409990071 19825260

[98] Leno VC Charman T Pickles A Jones CR Baird G Happe F. Callous–unemotional traits in adolescents with autism spectrum disorder. Brit J Psychiatry. (2015) 207:392–9. 10.1192/bjp.bp.114.159863 26382954 PMC4629071

[99] Lockwood PL Bird G Bridge M Viding E. (2013). Dissecting empathy: high levels of psychopathic and autistic traits are characterized by difficulties in different social information processing domains. Front Hum Neurosci. 7:760. 10.3389/fnhum.2013.00760 PMC3826592 24294197

[100] Martin-Key N Brown T Fairchild G. Empathic accuracy in male adolescents with conduct disorder and higher versus lower levels of callous-unemotional traits. J Abnor Child Psychol. (2017) 45:1385–97. 10.1007/s10802-016-0243-8 28032270 PMC5603649

[101] Harkins CM Handen BL Mazurek MO. The Impact of the Comorbidity of ASD. and, ADHD, on social impairment. J Autism Dev Disord. (2021). 10.1007/s10803-021-05150-1. [Epub ahead of print]. 34181141

[102] Foell J Brislin SJ Drislane LE Dziobek I Patrick CJ. Creation and validation of an english-language version of the multifaceted empathy test (MET). J Psychopathol Behav Assess. (2018) 40:431–9. 10.1007/s10862-018-9664-8

